# FMRP and CYFIP1 at the Synapse and Their Role in Psychiatric Vulnerability

**DOI:** 10.1159/000506858

**Published:** 2020-03-03

**Authors:** Nicholas E. Clifton, Kerrie L. Thomas, Lawrence S. Wilkinson, Jeremy Hall, Simon Trent

**Affiliations:** ^a^Neuroscience & Mental Health Research Institute, Cardiff University, Cardiff, United Kingdom; ^b^MRC Centre for Neuropsychiatric Genetics and Genomics, School of Medicine, Cardiff University, Cardiff, United Kingdom; ^c^School of Biosciences, Cardiff University, Cardiff, United Kingdom; ^d^School of Psychology, Cardiff University, Cardiff, United Kingdom; ^e^Division of Psychological Medicine and Clinical Neurosciences, School of Medicine, Cardiff University, Cardiff, United Kingdom; ^f^School of Life Sciences, Faculty of Natural Sciences, Keele University, Keele, United Kingdom

**Keywords:** Fragile-X mental retardation protein, Cytoplasmic FMRP-interacting protein 1, Fragile-X mental retardation protein targets, Synapse, Psychiatric disorders

## Abstract

There is increasing awareness of the role genetic risk variants have in mediating vulnerability to psychiatric disorders such as schizophrenia and autism. Many of these risk variants encode synaptic proteins, influencing biological pathways of the postsynaptic density and, ultimately, synaptic plasticity. Fragile-X mental retardation 1 (*FMR1*) and cytoplasmic fragile-X mental retardation protein (FMRP)-interacting protein 1 (*CYFIP1*) contain 2 such examples of highly penetrant risk variants and encode synaptic proteins with shared functional significance. In this review, we discuss the biological actions of FMRP and CYFIP1, including their regulation of (i) protein synthesis and specifically FMRP targets, (ii) dendritic and spine morphology, and (iii) forms of synaptic plasticity such as long-term depression. We draw upon a range of preclinical studies that have used genetic dosage models of *FMR1* and *CYFIP1* to determine their biological function. In parallel, we discuss how clinical studies of fragile X syndrome or 15q11.2 deletion patients have informed our understanding of FMRP and CYFIP1, and highlight the latest psychiatric genomic findings that continue to implicate FMRP and CYFIP1. Lastly, we assess the current limitations in our understanding of FMRP and CYFIP1 biology and how they must be addressed before mechanism-led therapeutic strategies can be developed for psychiatric disorders.

## The Synapse and Postsynaptic Density Proteins

Neurotransmission between presynaptic and postsynaptic terminals is the adaptive communication mechanism linking neurons and other cell types into neural circuits and networks, which form the basis of synaptic plasticity, cognition, and behaviour [1, 2]. The majority of excitatory, glutamatergic synapses in the mammalian brain are located at small dendritic protrusions, or spines [3], and contain a prominent assembly of proteins at the postsynaptic membrane known as the postsynaptic density (PSD) [4, 5]. Proteomic profiling of the PSD has revealed over 1,000 different proteins [6, 7, 8], many of which converge on the regulation of synaptic plasticity through biological pathways controlling protein synthesis, receptor trafficking, or structural rearrangements [9, 10, 11].

## Synaptic FMRP: Regulator of mRNA and Local Translation

One such synaptic protein is fragile-X mental retardation protein (FMRP), encoded by the *FMR1* gene (Xq27.3) [12] and the monogenic cause of neurodevelopmental disorder fragile X syndrome (FXS) [13]. *FMR1* mRNA is expressed in the neuronal cell body, developing and mature axons, dendrites and dendritic spines, and the nucleus [14, 15], but not across all neuronal populations [16, 17]. FMRP is an RNA-binding protein with multiple structural motifs for binding RNA (such as the K homology domain and arginine-glycine-rich, RGG, box) [18], capable of regulating the dendritic sequestering, and localization, of hundreds of target neuronal mRNAs [19, 20], either through direct interactions or via intermediary interactions with noncoding RNA [21, 22].

**Fig. 1:**
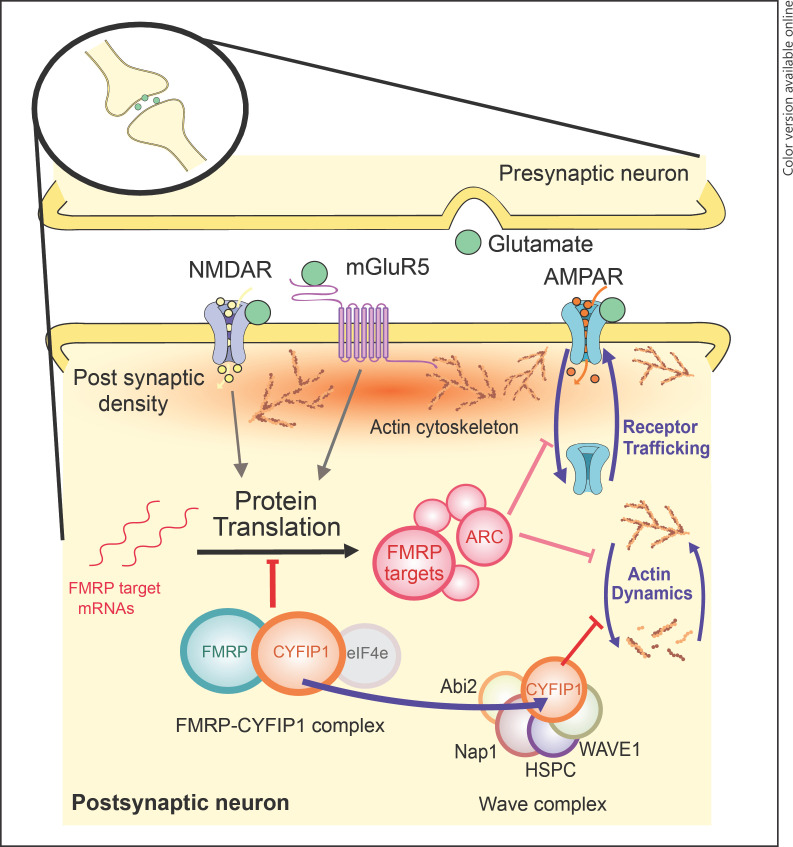
Biological roles of synaptic FMRP and CYFIP1 in postsynaptic neurons. FMRP plays a key role in negatively regulating the translation of hundreds of FMRP targets, including activity-regulated cytoskeleton associated (ARC), by forming a complex with CYFIP1, alongside the initiation factor eIF4e. The control of mRNA translation, and its repression by the CYFIP1-FMRP complex, is partly mediated through activation of upstream NMDA and mGluR5 receptors. FMRP targets such as ARC can drive changes in synaptic plasticity through regulation of α-Amino-3-hydroxy-5-methyl-4-isoxazolepropionic acid (AMPA) receptor trafficking/internalization and increasing actin cytoskeleton stability. Meanwhile, CYFIP1 can bind and inhibit the WAVE regulatory complex, thereby blocking the promotion of actin cytoskeleton rearrangements. Preclinical evidence suggests that under conditions of synaptic activation, CYFIP1 redistributes between the 2 main complexes, with greater association with the WAVE complex and a reciprocal decrease with the FMRP complex. FMRP, fragile-X mental retardation protein; CYFIP1, cytoplasmic FMRP-interacting protein; mGluR5, metabotropic glutamate receptor 5.

FMRP, its target mRNA, and other protein partners together form large messenger ribonucleoparticles (mRNPs) [23]. Within the mRNPs, FMRP plays a key role in the translational silencing of its target mRNA [24, 25, 26, 27], required during the transport of mRNA along dendrites [28], before synaptic activation results in the docking of the mRNPs to the spines and subsequent translation [29, 30]. FMRP specifically regulates the rate-limiting step of cap-dependent mRNA translation *initiation* by binding to the initiation factor eIF4E and FMRP-binding partner cytoplasmic FMRP-interacting protein 1 (CYFIP1) (see section “Synaptic CYFIP1: A Negative Regulator of Protein Synthesis and Cytoskeletal Dynamics” later) [31, 32], although initiation may also be regulated via FMRP ubiquitination or sumoylation [33, 34, 35]. FMRP also controls *elongation* stages of mRNA translation by stalling ribosomes on FMRP target transcripts [19, 24], although how FMRP switches between the regulation of initiation and elongation is currently unknown [36]. FMRP is, therefore, a critical mediator of local translation of mRNA targets, acting at both presynaptic and postsynaptic terminals [24, 37, 38]. Some of the key biological roles of FMRP at postsynaptic terminals are highlighted in Figure 1.

Beyond translational silencing, FMRP plays other biological roles [39], including RNA editing [40], regulation of mRNA target stability [41], and ion-channel binding [42, 43], collectively influencing calcium signalling [44], activity-dependent neurodevelopment [45], and the balance of excitatory/inhibitory circuits [46, 47]. The additional functions of FMRP might explain instances where FMRP does not appear to be a straightforward repressor of protein synthesis [48], perhaps most pertinently through FMRP's ability to influence the stability of a subset of mRNAs [22, 49].

## FMRP Targets

Considerable effort has been made to identify the mRNAs targeted by FMRP so that biological pathways affected by mutations in *FMR1* can be better predicted. Such studies have used immunoprecipitation followed by either microarray [50, 51] or high-throughput sequencing [19, 52] to determine FMRP-bound mRNAs. Due to varied methodology and tissues (as well as influence from type I and II errors), these lists of FMRP targets differ considerably [52, 53]. Therefore, precisely which mRNAs are bound by FMRP is uncertain and likely to be somewhat tissue-specific. Two studies using mouse cortical tissue and comparable methodology (high-throughput sequencing of RNA isolated by cross-linking immunoprecipitation) yielded highly overlapping results: 89% of mRNAs identified as FMRP targets by Darnell and colleagues [19] were also identified by Maurin et al. [52]. Still, only a small subset of the proposed targets have been validated [23, 49, 54, 55, 56, 57].

Gene ontology analyses of brain-derived FMRP targets confirm an overrepresentation of genes involved in functions related to synaptic activity, plasticity, development, and anatomy [19, 52, 58], consistent with studies of FMRP function. The proteins they encode include both presynaptic and postsynaptic components. Of these are subunits and interactors of receptor complexes considered central to synaptic plasticity phenotypes associated with FXS, chiefly the of metabotropic glutamate receptor 5 (mGluR5) and N-methyl-D-aspartate (NMDA) receptor signalling complexes [19]. The observation that FMRP binds some presynaptic proteins supports the evidence that FMRP regulates protein synthesis during axon development and synapse formation [59, 60, 61, 62].

Whilst studies of FMRP targets have identified probable interactions between FMRP and ribosomal mRNAs, further work is needed to determine whether the translation of these mRNAs is indeed repressed by FMRP within the regulatory complex together with CYFIP1 and eIF4E proteins in the cell [31, 32].

## Synaptic CYFIP1: A Negative Regulator of Protein Synthesis and Cytoskeletal Dynamics

CYFIP1 is a highly dynamic synaptic protein involved in numerous biological pathways through an array of protein-protein interactions (Fig. 1) [63]. Originally known as specifically Rac1-activated protein 1 (SRA-1) [64], CYFIP1 was later found to bind with FMRP [12, 65], forming a critical CYFIP1-FMRP complex at the synapse [31]. Specifically, FMRP-bound CYFIP1 acts as a non-canonical eIF4E-binding protein [31], thereby providing competition for the binding of eIF4E to the translation initiation complex (eIF4E-eIF4G) [66, 67]. Overall, it is this eIF4E-CYFIP1-FMRP complex, together with its target mRNA, that represses translation at dendritic and synaptic sites [31]. Upon synaptic activation via tropomyosin receptor kinase B or group I mGluRs, eIF4E is released from CYFIP1-FMRP and permits the translation of target mRNAs [31]. A subsequent study has implicated a mitogen-activated protein (MAP)-kinase-interacting kinase-dependent pathway in the release of the inhibitory CYFIP1-FMRP complex from target mRNA, via MAP-kinase-interacting kinase phosphorylation of CYFIP1, in the early phase of long-term potentiation (LTP), thereby permitting translation [68].

Aside from its role in regulating protein synthesis, CYFIP1 forms part of the ∼400-kDa heteropentameric WAVE regulatory complex, which also contains WAVE1/2/3, abl interactor-1/2 (ABI1/2), Nck-associated protein 1 (NCKAP1), and haematopoietic stem/progenitor cell protein 300 (HPSC300) components [69]. Without CYFIP1, the WAVE complex promotes actin cytoskeleton remodelling via the Arp2/3 complex [70, 71, 72], impacting on aspects of dendritic spine formation, stability, morphology, migration, and excitability [73]. The role of CYFIP1 is to maintain the WAVE complex in an inhibited state, until the GTPase, Rac1, causes the dissociation of CYFIP1 from the WAVE regulatory complex and allows actin remodelling to proceed via Arp2/3 [74].

CYFIP1 belongs to the two aforementioned complexes, FMRP and WAVE, in a mutually exclusive manner, skewed towards greater association with the WAVE complex, under basal conditions [74]. Notably, synaptic activation changes the protein conformation of CYFIP1, from globular to planar, and drives the distribution of CYFIP1 further towards the RAC1-WAVE complex, with a concomitant decrease in the eIF4E-CYFIP1 complex [71, 74, 75]. Therefore, CYFIP1 is a central molecular mediator that bridges the 2 processes of mRNA translation and actin dynamics, both essential for synaptic plasticity [76, 77, 78]. Other molecular roles for CYFIP1 are being explored, including its role presynaptically. For instance, presynaptic function is altered in the hippocampus of juvenile *Cyfip1* knockout (KO) mice, thought to derive from changes in presynaptic terminal size and enhanced vesicle release probability, and driven by dysregulation of the WAVE complex [79]. These findings closely align with previous findings in *cyfip1* mutant fly models that specifically found alterations in actin polymerization in presynaptic terminals [65, 80]. More recently, *Cyfip1* KO mice were found to have decreased myelination of callosal axons, alongside impaired presynaptic neurotransmission in the corpus callosum [81].

CYFIP1 has a closely related paralogue, CYFIP2, with over 88% amino acid identity [12]. Like CYFIP1, CYFIP2 is found at excitatory [82] and inhibitory [83] synapses, and binds both to the WAVE complex [63, 69] and to FMRP [12]. Interestingly, CYFIP2 additionally binds to FMRP-related proteins, FXR1P and FXR2P, while *CYFIP2* mRNA is a target of FMRP [19], implying a further layer of feedback between FMRP and the family of CYFIPs. However, the molecular redundancy between these paralogues is limited, given that the deletion of both copies of *CYFIP1* is embryonically lethal [74, 84]. Furthermore, CYFIP1, but not CYFIP2, has been consistently associated with neuropsychiatric disorders [82, 83; however, see 85].

## Psychiatric Disorders and the Synapse

Considerable evidence suggests that a wide range of neuropsychiatric disorders, such as FXS, autism spectrum disorders (ASDs), schizophrenia, intellectual disabilities (IDs), and bipolar disorder, exhibit convergent synaptic pathology [6, 32, 86, 87, 88, 89, 90, 91]. Synaptic dysfunction has been observed at several levels, including genetic alterations [92, 93, 94],aberrant proteins [95] and their translation [25, 96, 97, 98], molecular signalling pathways [99, 100, 101], spine morphology [102], aberrant synaptic plasticity [103, 104, 105], neurocircuitry, and connectivity [106]. These interrelated observations highlight impaired synaptic function as a common feature of several neuropsychiatric disorders [91, 94, 107].

In light of this view, and the biological importance of FMRP and CYFIP1 at the synapse (outlined in sections “Synaptic FMRP: Regulator of mRNA and Local Translation” and “Synaptic CYFIP1: A Negative Regulator of Protein Synthesis and Cytoskeletal Dynamics”), we will now consider the role of FMRP and CYFIP1 in the aetiology of psychiatric disorders, using data from human patient studies, especially psychiatric genomics, and preclinical models.

## FMRP and FMRP Targets in Psychiatric Disorders

### FXS Patients and Fmr1 KO Models

In humans, transcriptional silencing of the *FMR1* gene by a triplet repeat expansion (beyond 200 repeats, typically ∼800) in the 5-untranslated region of *FMR1*[108] leads to FXS [13, 109]. FXS patients display a broad range of abnormalities, including increased immaturity of dendritic spines [110, 111, 112], altered molecular signalling [23], increased levels of basal protein synthesis [113, 114], altered neuron and circuit excitability [115], structural and connectivity defects in brain networks [116], and a range of cognitive and behavioural phenotypes that overlap considerably with ID and ASD [117, 118, 119]. Indeed, FXS represents the single most common form of inherited ID with a prevalence of 1:4,000 males and 1:8,000 females [120] and the most common, single-gene cause of ASD [108, 117]. FMRP may also be involved in other neuropsychiatric disorders, beyond FXS and related ASDs, including schizophrenia and bipolar disorder [121, 122, 123, 124, 125].

The effects of *Fmr1* mutations have been interrogated preclinically for 25 years through the *Fmr1* KO mouse model [126] and, with the advent of modern gene-targeting technologies, the *Fmr1* KO rat model [127, 128]. Many of the features of human FXS have been recapitulated in *Fmr1* KO mouse and rat models, especially in 3 key areas: dendritic spine maturation [112, 127, 129, 130], elevated basal protein synthesis [127, 131, 132, 133], and behavioural/cognitive phenotypes, including ASD-like abnormalities [134], abnormalities in social interaction and interest [135], social anxiety [136], and reduced behavioural flexibility/reversal learning in a variety of tasks [127, 137, 138, 139, 140, 141].

In addition to heightened global protein synthesis, *Fmr1* KO rodents display a lack of mGluR-dependent translational control, which results in an elevated protein synthesis-dependent form of synaptic plasticity, known as mGluR-mediated long-term depression [127, 142, 143, 144, 145]. Increased mGluR-dependent translation is thought to occur through excessive activation of the mGluR5 subtype, given that reductions in mGluR5 expression [132], or the mGluR5 antagonist 2-Methyl-6-(phenylethynyl)pyridine (MPEP)[46, 146], can rescue several *Fmr1* KO phenotypes. The altered mGluR5 signalling in the absence of *Fmr1* appears to be mediated through the preferential interaction of mGluR5 with activity-dependent isoforms of Homer1 over constitutive Homer proteins [147, 148].

The deletion of *Fmr1* results in the loss of the repressive eIF4E-Cyfip1-FMRP complex, which de-represses the initiation complex, eIF4F, required for cap-dependent translation initiation of FMRP targets [149]. It was shown that an inhibitor of the eIF4F complex, which creates free eIF4E, increases the abundance of the eIF4E-CYFIP1-FMRP complex (with a parallel decrease in the CYFIP1-WAVE complex) in *Fmr1* KO mice, and the restoration of this imbalance rescues spine and memory deficits in these animals [150]. Hence, studies of the *Fmr1* KO rodent model have illuminated a variety of molecular mechanisms relevant to FXS, especially those pertinent to the regulation of protein synthesis, and may provide biological targets for therapeutic intervention [24], complementing ongoing clinical trials in human FXS patients [151, 152].

### FMRP and FMRP Targets in Psychiatric Genomic Studies

Beyond repeat expansions in the *FMR1* gene, a number of rare pathogenic point mutations have been reported that cause developmental delay and ID reminiscent of FXS [153, 154, 155, 156, 157]. Further evidence suggests that mutations in the autosomal homolog *FXR2* gene might also contribute to ID [158, 159, 160]. Whilst variants affecting the related *FXR1* gene confer risk to schizophrenia, bipolar disorder, and autism [161, 162, 163, 164, 165], the genetic link between *FMR1* and psychiatric disorders derives from enrichment of association within the gene targets of FMRP (among which the fragile-X family genes themselves are included).

A set of FMRP target mRNAs derived from a study of mouse cortical polyribosomes [19] have been recurrently highlighted in the literature due to their enrichment for genes associated with an array of psychiatric disorders. Through large-scale genome-wide association studies, these 842 FMRP targets have been shown to be genetically associated with schizophrenia [161, 162], autism [166], major depressive disorder [167], and bipolar disorder [58]. In addition to the risk conferred from common variation, this gene set is enriched for rare variants from patients with schizophrenia [168, 169, 170, 171], autism [172], and bipolar disorder [173]; de novo variants from patients with schizophrenia [174] and autism [175, 176, 177]; and to a lesser extent copy number variants from patients with schizophrenia [178, 179, 180]. The convergence of risk from multiple different types of genetic variants forms a strong evidence base, implicating this gene set in psychiatric pathology. Conversely, FMRP targets derived from a study of human embryonic kidney cells [51] do not appear to be associated with psychiatric disorders [166, 168], highlighting the tissue specificity of these relationships.

Brain FMRP targets overlap considerably with other gene sets associated with psychiatric disorders, such as genes encoding PSD proteins and those involved in calcium signalling, synaptic plasticity, learning, and memory [19, 52, 58, 181]. However, despite these overlaps, the enrichment of brain FMRP targets for association with psychiatric disorders is independent [58, 162, 168] and proportional to the confidence of binding by FMRP [58]. Moreover, in many instances, it appears that FMRP targets capture subsets of these other gene sets in which genetic association is concentrated [58]. Hence, this set of genes locally regulated by FMRP during plasticity and development at the synapse may represent a collection of biological pathways important for the manifestation of a range of psychiatric disorders.

## CYFIP1 in Psychiatric Disorders

### 15q11.2 Copy Number Variants and Cyfip1 Dosage Models

The proximal long arm of human chromosome 15 (15q11.2–13.3) is a region of numerous low-copy repeats that can lead to aberrant meiotic chromosomal rearrangements. These result in deletions or duplications of sections of DNA, known as copy number variants (CNVs), and occur at any of 5 common breakpoints (BP1–BP5) on chromosome 15 [182]. Neurodevelopmental psychiatric disorders Prader-Willi syndrome and Angelman syndrome are caused by deletions of paternal or maternal origin, respectively, and occur as either large deletions (type I, between BP1 and BP3) or smaller deletions (type II, between BP2 and BP3). Meanwhile, *CYFIP1* is cytogenetically positioned in the non-imprinted 500-kb region between BP1 and BP2 on chromosome 15 (15q11.2 interval), along with 3 additional genes: non-imprinted in Prader-Willi/Angelman 1 (*NIPA1*) and 2 (*NIPA2*), and tubulin gamma complex-associated protein (*TUBGCP5*) [183]. The 15q11.2 chromosomal region was first implicated with neurodevelopmental psychiatric disorders through the observation that type I deleted Prader-Willi syndrome or Angelman syndrome patients, who lack the 15q11.2 interval, had more severe behavioural phenotypes than type II deleted patients, in whom the 15q11.2 interval is intact [184, 185]. Later, patients were identified with deletions and duplications between BP1 and BP2, which specifically flanked the 15q11.2 interval itself [182].

Deletions or duplications of 15q11.2 are present in 1 of 100 people who present for genetic screening, whilst incidence in the general population is likely to be nearer 1 in 500 people [186]. The CNV causes patients to display language/motor deficits or delays, behavioural problems, autism, and seizures [187, 188, 189], with deletions being the most impactful on cognition [187] and referred to as Burnside-Butler syndrome [182, 186]. It was recently observed that 15q11.2 deletion patients have structural and functional changes in the brain that likely relate to the accompanying cognitive phenotypes, including a smaller left fusiform gyrus and altered activation in the left fusiform and the left angular gyri using functional magnetic resonance imaging [190]. In subsequent diffusion tensor imaging studies, 15q11.2 deletion carriers show increased fractional anisotropy [191], indicating alterations in the white matter microstructure [192]. In keeping with these findings, white matter changes in 15q11.2 deletion patients closely mirror the phenotypes of FXS patients [193], suggesting a common pathogenic pathway derived from disruption of CYFIP1-FMRP complexes. Although the 15q11.2 deletion is not fully penetrant, as a significant proportion of the general population are healthy carriers with no overt phenotypes [194], it is likely that subclinical cognitive phenotypes exist even in these “healthy” carriers [195].

Among the genes located within the 15q11.2 locus, *CYFIP1* is widely regarded as the most likely to confer the biological and behavioural phenotypes associated with 15q11.2 BP1–BP2 CNVs [84, 191]. This is due, in part, to its known functional association with the FXS-relevant protein FMRP (see sections “Synaptic FMRP: Regulator of mRNA and Local Translation,” “FMRP Targets,” “Synaptic CYFIP1: A Negative Regulator of Protein Synthesis and Cytoskeletal Dynamics,” and “FMRP and FMRP Targets in Psychiatric Disorders”) [31, 74]. Furthermore, the expression of CYFIP1 and components of the WAVE complex is disrupted in patients carrying 15q11.2 deletions [196]; iPSCs derived from these patients exhibit cellular phenotypes mediated by the CYFIP1-WAVE complex [197]; and the knockdown of CYFIP1, specifically, in human progenitor cells alters cytoskeletal remodelling [198]. However, the biological roles of the three remaining genes within the 15q11.2 interval requires further delineation, as, like *CYFIP1*, they are all expressed in the central nervous system and their expression is altered in patients with 15q11.2 CNVs [199].

Great strides have been made in understanding the consequences of altered *Cyfip1* dosage through a variety of in vitro and in vivo rodent preclinical models. For instance, the heterozygous deletion of *Cyfip1* in mice results in changes in dendritic and spine morphology [74, 82], which are similarly observed in a forebrain-specific conditional homozygous KO model [83], whilst the overexpression of *Cyfip1* also impinges on dendrite and spine morphology [82, 200]. Meanwhile, *Cyfip1* appears to affect protein synthesis under basal and activity-dependent conditions. The knockdown of *Cyfip1* in cortical neurons in vitro increases the translation of FMRP target, activity-regulated cytoskeleton associated (ARC), under basal conditions and also ablates the activity-dependent translation of ARC, using brain-derived neurotrophic factor treatment to mimic synaptic activation [74]. Similar findings were reported in vivo using *Cyfip1* heterozygous KO mice, whereby brain-derived neurotrophic factor treatment was insufficient to release the Cyfip1-FMRP complex from eIF4E, preventing the formation of the eIF4F complex, which subsequently prevented activity-dependent translation of ARC protein [68].

Measures of synaptic plasticity in preclinical models of altered *Cyfip1* dosage have revealed elevated levels of mGluR-mediated long-term depression, which become disassociated from mRNA translation pathways [84] − findings that are reminiscent of *Fmr1* KO rodent models [127, 144]. Overexpressing *Cyfip1* in CA1 hippocampal neurons can lead to increased excitatory neurotransmission, and a concomitant decrease in gamma aminobutyric acid (GABA)ergic neurotransmission at inhibitory synapses, shifting the overall excitation/inhibition balance towards excessive excitation [83]. The same study also showed that the conditional, homozygous KO of *Cyfip1* in CA1 hippocampal neurons increased inhibitory GABAergic neurotransmission, along with increased expression of GABA receptors, suggesting a shift of excitation/inhibition balance towards greater inhibition [83]. However, in the haploinsufficient *Cyfip1* mouse model, which better models the reduced dosage of *CYFIP1* in 15q11.2 deletion patients, GABAergic signalling remains unaltered in the hippocampal dentate gyrus [201].

Brain connectivity and white matter architecture appear to be especially sensitive to reduced *Cyfip1* dosage. In *Cyfip1* heterozygous KO mice, bilateral connectivity was shown to be reduced across multiple brain regions using resting-state functional magnetic resonance imaging [81]. These alterations were likely due to changes in corpus callosal white matter architecture, measured by (i) a decrease in fractional anisotropy using diffusion tensor imaging and (ii) altered levels of myelination and presynaptic function. Furthermore, many of the white matter phenotypes, including decreased fractional anisotropy, were mirrored in a comparable rat model of *Cyfip1* haploinsufficiency [202]. However, it is currently unclear why fractional anisotropy might be decreased in rodent models of reduced *Cyfip1* dosage but increased in 15q11.2 deletion patients. This will require further study and may alter our current perception of the effect of CNVs of the 15q11.2 interval.

**Fig. 2:**
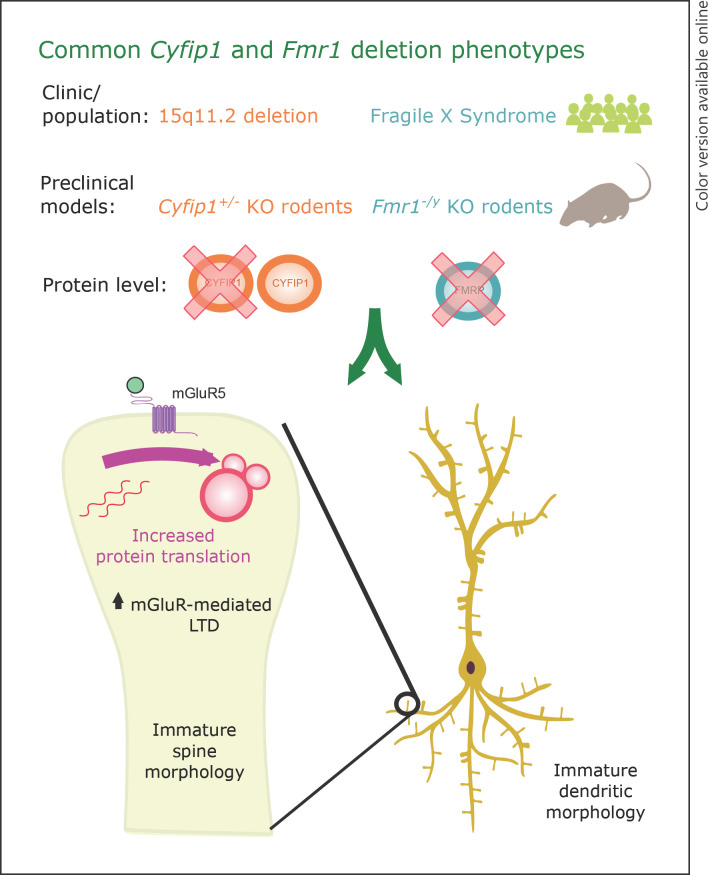
Core set of overlapping phenotypes from preclinical *Fmr1* and *Cyfip1* deletion models. Rodent models of *Fmr1* deletion (*Fmr1−/y*, whereby the single X-linked copy of *Fmr1* is deleted in males) or heterozygous *Cyfip1* deletion (*Cyfip1+/−*) mirror clinical populations with FXS and 15q11.2 CNV deletions, respectively. Moreover, these 2 rodent models share a core set of functionally related neurobiological phenotypes, including (i) altered spine and dendritic morphology, (ii) dysregulated protein translation and (iii) elevated long-term depression. Further work is required to fully delineate the consequences of *Fmr1* and *Cyfip1* deletion, and characterize the similarities. FXS, fragile X syndrome; CNV, copy number variant.

In vivo models of altered *Cyfip1* dosage also offer the chance to thoroughly assess changes in behaviour and cognition, prominent features in 15q11.2 deletion (and duplication) patients. Bozdagi and colleagues [84] were the first to behaviourally assess *Cyfip1* haploinsufficient mice, and found many aspects of spatial and fear learning and memory to be intact, with the exception of a rapid loss of extinction memory assessed using the inhibitory avoidance paradigm. Subsequent analysis of *Cyfip1* heterozygous KO mice and rats has shown specific deficits in motor learning [81, 203], sensorimotor gating measured by prepulse inhibition [81], and behavioural flexibility [202]. Meanwhile, the overexpression of *Cyfip1* results in cellular phenotypes, particularly at the dendritic level [200], but appears to have little effect on behaviour and cognition, with the exception of exaggerated fear responses [204]. Overall, there is accumulating evidence that altering the dosage of *Cyfip1* in preclinical models leads to profound alterations in cellular and plasticity phenotypes, alongside mild behavioural phenotypes, many of which not only overlap with FXS and the *Fmr1* KO model (Fig. 2), but also closely match the key clinical phenotypes of patients with chromosomal deletions (and duplications) of the *CYFIP1*-containing 15q11.2 interval.

### CYFIP1 Variants in Psychiatric Genomic Studies

Genomics studies in psychiatric populations have implicated the 15q11.2 BP1–BP2 deletion with a wide range of psychiatric, neurodevelopmental disorders, including a 2- to 4-fold increased risk for schizophrenia [205, 206], a finding that has been replicated in many subsequent studies [92, 179, 207, 208, 209, 210]. Additionally, 15q11.2 deletions, and duplications, predispose individuals to a 5-fold risk of epilepsy [211], developmental and ID [212, 213, 214], attention deficit hyperactivity disorder [215], major depression [216], and autism [187, 217] [for further review, see 182, 186]. Meanwhile, common variants in *CYFIP1* have been reported to increase the risk for ASD [218, 219]. Consistent with the genetic findings, proteomic analysis of prefrontal cortex post-mortem tissue from schizophrenia patients revealed altered levels of CYFIP1 and other proteins belonging to protein synthesis pathways [220].

The relevance of CYFIP1 to schizophrenia becomes especially apparent when considered in the wider context of its biological actions within protein complexes. CYFIP1 is involved in the regulation of ARC protein and ARC-related genes, sometimes referred to as the “ARC complex” (a gene ontology-based complex). *CYFIP1* was first associated with schizophrenia in studies that showed an enrichment of the ARC complex (containing 25 genes, of which *CYFIP1* is one) in de novo CNV deletions from patients with schizophrenia [92]. The genetic association of this ARC complex with schizophrenia has subsequently been confirmed by exome sequencing studies that assessed single nucleotide variants (SNVs) and indels [168, 174] and larger studies of CNV deletions [178, 179]. Furthermore, the genetic association with schizophrenia of FMRP targets (section “FMRP and FMRP Targets in Psychiatric Genomic Studies”), which are regulated by the CYFIP1-FMRP complex, lends additional evidence to the relevance of CYFIP1 to schizophrenia.

## Summary of Findings and Future Directions

FMRP and CYFIP1 are hubs for several biological pathways critical to synaptic plasticity. From preclinical models, we know that reduced expression of either CYFIP1 or FMRP results in a set of core phenotypes: altered spine and dendritic morphology, dysregulated protein synthesis, and elevated long-term depression. A further layer of complexity is added when it is considered that the concerted action of FMRP and CYFIP1, as part of the CYFIP-FMRP complex, represses the translation of hundreds of FMRP targets, likely influencing multiple downstream pathways. The importance of this system to synaptic function is recurrently highlighted by genetic studies demonstrating the risk conferred to psychiatric disorders by variants affecting genes encoding CYFIP1, FMRP, and their targets.

Nevertheless, there are many questions that still surround the biology of FMRP, CYFIP1, and FMRP targets in health and disease. For example, whilst FMRP synaptic biology is well-characterized and preclinical techniques can reverse disorder-relevant phenotypes [132, 151], attempts to move these therapies into the clinic have been largely ineffective [152]. This suggests that further mechanistic insights into the actions of FMRP are needed, alongside further refinement of therapeutic targets and/or strategies. Similarly, whilst FMRP targets are a disease-relevant group of mRNAs, their precise identity and biological function remain underexplored. Meanwhile, the study of CYFIP1 has seen unprecedented advances in recent years, revealing an extensive array of synaptic roles, far beyond its initial characterization as a binding partner to FMRP. Despite the rapid expansion of CYFIP1 studies, many fundamental questions remain and can be addressed in future studies, aided by advances in RNA sequencing, genetic-editing, and proteomic technologies. Whilst extensively characterized, it is also worth noting that the behavioural phenotypes in models of *Fmr1* and *Cyfip1* deletion are only broadly similar, and in some cases diametrically opposed [221]. These behavioural discrepancies could reflect the diversity of biological function, but might also derive from highly transient and localized interactions between these two proteins.

Penetrant risk variants affecting this biological pathway increase psychiatric vulnerability to a range of psychiatric disorders. For example, CNVs affecting *CYFIP1* predispose carriers to increased risk for schizophrenia (mainly 15q11.2 deletions), autism, and ID (mainly 15q11.2 duplications), and likewise *FMR1* deletions predispose carriers to autism and ID. These apparently pleiotropic effects might suggest that the categorical nature of diagnoses for psychiatric disorders needs to be fundamentally re-evaluated. Indeed, at the clinic, there are many common patient symptoms that span across diagnostic categories, and patients often present with comorbidities. The genomic findings point towards a continuum of causality, whereby common biological mechanisms, influenced by a range of convergent genetic factors, span across the traditional diagnostic boundaries of psychiatric disorders. The highly tractable mechanism of CYFIP1-FMRP and the regulation of ARC are one such biological pathway, offering a unique entry point for continued study and phenotypic rescue. Future development of novel mechanism-based therapeutic approaches will be vital to meet the ever-growing need to treat these common, yet debilitating, psychiatric disorders.

## Disclosure Statement

The authors have no conflicts of interest to declare.

## Funding Sources

The work was supported by a Wellcome Trust Strategic Award (DEFINE, 100202/Z/12/Z) and a Medical Research Council Centre Grant (GO801418). S.T. and N.E.C. were supported by Cardiff University Neuroscience and Mental Health Research Institute Fellowships. S.T. has been additionally supported by startup funds associated with a new appointment as lecturer at Keele University.

## Author Contributions

The review was conceived by S.T. and written by S.T. and N.E.C.; K.L.T., L.S.W., and J.H. provided additional text, comments, and support. Figures were created by S.T. and N.E.C.
